# Imaging-based vascular-related biomarkers for early detection of acetaminophen-induced liver injury

**DOI:** 10.7150/thno.44900

**Published:** 2020-05-18

**Authors:** Haolu Wang, Leslie J. Burke, Jatin Patel, Brian WC. Tse, Kim R. Bridle, Victoria C. Cogger, Xinxing Li, Xin Liu, Haotian Yang, Darrell H. G. Crawford, Michael S. Roberts, Wenchao Gao, Xiaowen Liang

**Affiliations:** 1 The University of Queensland Diamantina Institute, The University of Queensland, Brisbane, QLD, 4102, Australia; 2 Gallipoli Medical Research Institute, Greenslopes Private Hospital, Brisbane, QLD, 4120, Australia; 3 Faculty of Medicine, The University of Queensland, Brisbane, QLD, 4072, Australia; 4 Department of Biliary-pancreatic Surgery, Ren Ji Hospital, School of Medicine, Shanghai Jiao Tong University, Shanghai, 200127, China.; 5 Preclinical Imaging Facility, Translational Research Institute, Brisbane, QLD, 4102, Australia; 6 The University of Sydney, Concord Hospital, Concord, NSW, 2139, Australia; 7 Department of General Surgery, Changzheng Hospital, The Second Military Medical University, Shanghai, 200003, China

**Keywords:** Acetaminophen, Liver Injury, Endothelial cells, Vascular-related biomarkers, Ultrasonography

## Abstract

Acetaminophen (APAP) is the foremost cause of drug-induced liver injury in the Western world. Most studies of APAP hepatotoxicity have focused on the hepatocellular injury, but current hepatocyte-related biomarkers have delayed presentation time and a lack of sensitivity. APAP overdose can induce hepatic microvascular congestion, which importantly precedes the injury of hepatocytes. However, the underlying molecular mechanisms remain unclear. It is imperative to discover and validate sensitive and specific translational biomarkers of APAP-induced liver injury.

**Methods**: In this study, we assessed APAP toxicity in sinusoidal endothelial cells and hepatocytes in mice treated with overdose APAP at different time points. The underlying mechanisms of APAP overdose induced sinusoidal endothelial cell injury were investigated by RT^2^ Profiler PCR arrays. The impact of APAP overdose on endothelial cell function was assessed by pseudovessel formation of endothelial cells in 2D Matrigel and *in vivo* hepatic vascular integrity using multiphoton microscopy. Finally, the effects of APAP overdose on oxygen levels in the liver and hepatic microcirculation were evaluated by contrast enhanced ultrasonography. Potential imaging-based vascular-related markers for early detection of APAP induced liver injury were assessed.

**Results**: Our study confirmed that hepatic endothelial cells are an early and direct target for APAP hepatotoxicity. ICAM1-related cellular adhesion pathways played a prominent role in APAP-induced endothelial cell injury, which was further validated in primary human sinusoidal endothelial cells and human livers after APAP overdose. APAP overdose impacted pseudovessel formation of endothelial cells and *in vivo* hepatic vascular integrity. Use of ultrasound to detect APAP-induced liver injury demonstrated that mean transit time, an imaging-based vascular-related biomarker, was more sensitive and precise for early detection of APAP hepatotoxicity and monitoring the treatment response in comparison with a conventional blood-based biomarker.

**Conclusion**: Imaging-based vascular-related biomarkers can identify early and mild liver injury induced by APAP overdose. With further development, such biomarkers may improve the assessment of liver injury and the efficacy of clinical decision-making, which can be extended to other microvascular dysfunction of deep organs.

## Introduction

Acetaminophen (APAP) is one of the most commonly-used drugs for analgesia and fever reduction 1. However, APAP hepatotoxicity remains one of the leading causes of liver injury, with about 26,000 hospitalizations and 500 deaths each year in the US alone 2, 3. Current APAP toxicity assessment and the decision to start treatment are predominantly based on the ingested dose of APAP and a timed blood APAP concentration, and measurement of liver enzyme levels such as alanine aminotransaminase (ALT) 2, 3. Clinical uncertainty regarding the presence of hepatocyte injury prevents treatment being individualized, resulting in patients either being over treated with a time-consuming potentially harmful antidote or being undertreated with the attendant risk of serious liver injury 2. Hence, novel biomarkers or panels of biomarkers are required for evaluating the severity and response to treatment of APAP induced liver injury at the earliest possible time.

Most investigations on biomarker development for APAP-induced liver injury have focussed on biomarkers based on mechanistic processes involved in hepatocyte damage 4, 5. Recent studies have examined the potential use of new blood-based biomarkers such as microRNA-122, mitochondrial DNA, and keratin-18 from hepatocytes in human and murine models 2, 6. We have also reported the use of the hepatic optical oxidative stress index for monitoring early treatment response 7. However, APAP can induce hepatic microvascular injury and congestion, which importantly precedes hepatocyte injury. APAP-induced hepatic congestion in humans was first reported in 1969 8. Microvascular congestion has been found to precede hepatocyte injury 9-11. APAP is also toxic to sinusoidal endothelial cells 9, resulting in collapse of the sinusoidal wall and the infiltration of blood elements into the space of Disse 10, 11. Despite the evidence that liver sinusoidal endothelial cells are an early target of APAP injury to the liver 12, the mechanisms of APAP-induced microvascular injury remain to be elucidated. Furthermore, there are no vascular-related biomarkers for liver injury, mainly due to the absence of suitable technologies for detecting subtle alterations in hepatic microcirculation.

Only recently, advances in ultrasonography have led to the provision of non-invasive and quantitative imaging data on the hepatic microcirculation for diagnosis and evaluation of response to treatment in patients or animals with liver cirrhosis 13, fatty liver disease 14, and liver ischemia reperfusion injury 15. Two ultrasound-based modalities that have potential for reporting disease progression and evaluating treatment response are photoacoustic (PA) imaging 16, 17 and contrast-enhanced ultrasonography (CEU) 18-20. PA imaging is a hybrid modality whereby short near infrared NIR laser pulses are used to generate thermoacoustic waves in tissues containing light-absorbing endogenous or exogenous contrast agents 21, 22. PA imaging allows parallel collection of anatomical and functional information of tissue oxygen saturation which often changes in diseased conditions 23, 24. CEU is another imaging technique with gas-filled microbubbles as contrast agents that infuse into the intravascular space of the body, which enables assessment of tissue perfusion and is already clinically approved 25-27. Two perfusion models are commonly used to represent either bolus kinetics or replenishment kinetics following microbubble destruction. Microbubble velocity corresponds to the blood flow velocity 13. mTT is the average amount of time that it takes blood to transit through a given volume of an organ. In a destruction replenishment perfusion model, the amplitude of the plateau plus offset amplitude represents the relative blood volume in a quantified region. The product of microbubbles velocity, mTT, and tissue blood volume reflects regional perfusion 13. All these imaging-based vascular-related markers have the potential for early detection of APAP-induced liver injury.

Therefore, in present study, we investigated the mechanisms of APAP-induced hepatic microvascular injury in mice. We demonstrate that imaging-based vascular-related markers detected by ultrasonography are more sensitive and precise than conventional blood-based biomarkers for the early detection of APAP hepatotoxicity and for monitoring treatment response.

## Materials and Methods

**Animals.** Male BALB/c mice (8 weeks old) were purchased from the Animal Resource Centre (Perth, Western Australia). All animal procedures were approved by the Animal Ethics Committee of the University of Queensland (SOM/TRI/211/15) and were carried out according to Australian Code for the Care and Use of Animals for Scientific Purposes 8^th^ edition. APAP (Sigma-Aldrich) was dissolved in warm saline at the concentration of 15 mg/ml. The animals received a single gavage of 500 mg/kg APAP for 2 h or 12 h. Control animals were gavaged with saline. For n-acetylcysteine (NAC) treatment group (labelled with APAP 12h + NAC in all figures), animals were treated with 300 mg/kg NAC (Sigma-Aldrich, St Louis, MO, USA) by intraperitoneal injection 1 h after APAP administration and animals were assessed 12 h after APAP administration (n = 5 for each group), according to the reported procedure 28.

**Histology and TUNEL assay.** Liver tissues were collected, fixed, and embedded in paraffin. Paraffin sections were stained with hematoxylin and eosin for histological examination. TUNEL *in situ* assay was used for visualization of DNA stand breaks and apoptosis.

**TEM imaging of liver tissues.** Liver tissues were cut into approximately 1 mm^3^ cubes and fixed with 2.5% glutaraldehyde. Samples were embedded with epoxy resin, sectioned and imaged using a Philips CM10 electron microscope.

**Serum biochemical measurements.** A blood sample was collected and the plasma concentration of ALT was measured using a Hitachi 747 analyser (Hitachi Ltd., Tokyo, Japan) at Pathology Queensland, Princess Alexandra Hospital, Brisbane, Australia.

**PCR array.** Human umbilical vein endothelial cells, HUVECs, and human hepatic endothelial cells SK-HEP-1, were cultured in Endothelial Basal Medium (EBM-2) supplemented with EGM-2 SingleQuot supplements (Lonza, Basel, Switzerland) or DMEM containing 10% Fetal Bovine serum, respectively at 37 °C in 5% CO_2_.

Cells (1-2 X10^5^) were seeded in triplicate into 12 well dishes and when 80% confluent, were treated with APAP (Sigma Chemical Company, 20mM) for 6 h. Cells were harvested into RLT buffer (Qiagen, Hilden, Germany). RNA was extracted using a RNeasy Micro Plus Kit (Qiagen, Hilden, Germany). Total RNA was quantified using a NanoDrop Spectrophotometer (Thermo Scientific). Reverse transcription was performed with a RT^2^ First Strand Kit (Qiagen, Hilden, Germany) and 250 ng total RNA. qPCR was carried out using RT² SYBR Green ROX qPCR Mastermix (Qiagen, Hilden, Germany) and a RT^2^ Profiler™ Human Endothelial Cell Biology Array (PAHS-015Z: Qiagen, Hilden, Germany) that contains 84 genes related to endothelial cell biology using the ABI Viia7 Real-Time PCR system (Thermofisher, Waltham, MA, USA).

Data was analysed using the RT^2^ Profiler PCR Array Data Analysis Webportal at GeneGlobe (http://www.qiagen.com/geneglobe). CT values were normalized using the ΔCt method based on an automatic selection from the house keeping gene panel of reference genes.

Genes that exhibited more than 1.5 fold change in expression from the untreated cells, with a p-value of ≤ 0.05, were further analyzed using Ingenuity Pathway Analysis (IPA) software (Qiagen, Hildan, Germany) to determine pathway enrichment and cellular context of the differentially expressed genes.

**Patients and database.** Samples of publicly available human datasets of APAP overdose from the Gene Expression Omnibus, GSE74000 were analysed using GEO2R at https://www.ncbi.nlm.nih.gov/geo/ 28, 29. GSE74000 contains gene expression microarray data of liver biopsies from healthy humans (GSM1907918 and GSM1907919) and patients APAP-induced acute liver failure (Samples GSM1907915, GSM1907916 and GSM1907917). These data address differential gene expression in severe APAP-induced liver injury and were normalised and deposited by the original authors. Fold change (2^log-FC) and adjusted p-values were calculated relative to control samples.

**Validation of relative gene expression.** Mouse liver tissues were disrupted and lysed in RLT buffer containing 1% β-mercaptoethanol using a TissueLyser LT (Qiagen, Hilden, Germany). Lysates were further homogenised using a QIAshredder spin column (Qiagen, Hilden, Germany) followed by centrifugation to remove insoluble material. Primary human hepatic sinusoidal endothelial cells (Zenbio) were cultured in endothelial cell growth medium-1 and treated by 20 mM APAP for 6 h. Total RNA was extracted using an RNeasy Plus Mini Kit (Qiagen, Hilden, Germany). cDNA was prepared from purified RNA using a SensiFAST cDNA Synthesis Kit (Bioline, Memphis, TN). qRT-PCR was performed using a ViiA 7 Real-Time PCR System. Gene expression relative to HPRT (mouse liver samples, SK-HEP-1 cells and HSE cells) or B2M (beta-2-microglobulin) (HUVEC cells) was normalised to untreated samples.

**Flow cytometric analysis for the specific cell marker.** Liver tissues were dissociated into single cells in phosphate-buffered saline/bovine serum albumin/ethylenediaminetetraacetic acid. Dissociated single cells were further incubated with antibody combinations for multi-parameter flow acquisition and analysis. A Gallios flow cytometer was used for sample acquisition, whereas unbiased data analyses were performed with Kaluza analysis software (Beckman-Coulter, Brea, CA, USA). For each sample, 300,000 single cells were analyzed. The following combinations of antibodies were used to assess the endothelial population: Rat anti-mouse VE-Cadherin FITC, VEGFR2 PE, CD31 PE-Cy7, CD34 Alexa647, and Lineage PacBlue (Becton Dickinson, Franklin Lakes, NJ, USA).

### Tube formation in 2D Matrigel

Pseudovessels were formed by SK-HEP-1 cells mixed with green fluorescent protein (GFP) human endothelial colony forming cells (ECFC) in 2D Matrigel or HUVEC mixed with ECFC. SK-HEP-1 and HUVEC cells were stained with CellTracker™ Red CMTPX Dye (Thermofisher, C34552) at 37 °C for 1 h. 2.5x10^4^ SK-HEP-1 cells and 2.5x10^4^ ECFC cells were then mixed and seeded into 8-well chamber slide (Cellvis, C8-1-N) coated with 100 μl of Matrigel (CORNING, 356237) diluted in 100 μl of EGM-2 after 1hr of polymerization at 37 °C. Tube formation was monitored by Olympus IX81 Live Imaging Microscope. Cell were maintained at 37 °C, 5% CO_2_ during the whole imaging period. Time-lapse acquisitions were performed every 30 min up to 12 h by FITC and Cy3 channels to capture the signals of ECFC cells and SK-HEP-1 and HUVEC cells, respectively.

**Multiphoton microscopy imaging of liver tissues.** The settings of the multiphoton microscope were as previously described and established 30. A DermaInspect system (JenLab GmbH, Jena, Germany) equipped with an ultrashort pulsed mode-locked tunable Titanium: Sapphire laser (Mai Tai, USA) was used for imaging of mouse livers. Rhodamine B isothiocyanate/dextran 70 kDa (DXR70) was intravenously injected to mice and DXR70 fluorescence was detected at the excitation wavelength of 900 nm, which can be differentiated from autofluorescence of the liver. A bandpass filter was employed to detect fluorescence emission in the spectral range of 350 to 650 nm. High (40×, Zeiss) magnification objectives were used with the laser power set at 15 mW. DXR70 (250 μg/ml, 100 μl) was injected intravenously for labelling hepatic sinusoids.

***In vivo* ultrasound-based imaging.** All PA and CEU imaging were performed on a Vevo 2100 LAZR imaging station (Visualsonics, Toronto, Canada). Depilated mice were anaesthetized by 1.5% isoflurane inhalation (1 L oxygen / min) delivered via a nose cone and kept warm on a heated stage. B-mode images were used to locate the same position of the left liver lobe on every mouse for functional analysis.

**PA imaging and analysis.** Photoacoustic data were acquired using a LZ400 linear array transducer, which has a centre frequency of 30 MHz. The laser energy was calibrated and optimised using an in-built energy meter prior to measurements. All mice were scanned with their surface 10 mm below the transducer, and the PA signals were amplified 40 dB. Tissue oxygen saturation can be quantified based on differences in the absorbance spectrum between oxygenated haemoglobin (Hb_oxy_) and deoxygenated haemoglobin (Hb_deoxy_). For oxygen saturation measurements, dual-wavelength photoacoustic imaging at 750 nm and 850 nm was performed. The relative amounts of Hb_oxy_ and Hb_deoxy_ were calculated using the OxyZated Tool within Vevolab software (Visualsonics, Toronto, Canada). Oxygen saturation was defined as [100% × Hb_oxy_] / [Hb_oxy_ + Hb_deoxy_]. The threshold for Hb was set at 20% of maximal intensity, a reasonable value determined empirically 23.

**CEU imaging and analysis.** Non-linear CEU imaging was performed using a MS250S transducer at a frequency of 18 MHz, power of 4%, and contrast gain of 30 dB, following intravenous bolus infusion of 50 ul of non-targeted microbubbles (Visualsonics, Toronto, Canada). Hepatic microvasculature perfusion kinetics were assessed by two models conducted in sequence; (1) bolus perfusion model, then (2) destruction-replenishment perfusion model 31. Bolus perfusion imaging is based on the wash-in and wash-out kinetics of microbubbles after bolus injection 31. For this, cine loop recordings at 11 frames/ sec in contrast mode commenced immediately after tail vein injection of a single bolus of microbubbles at a consistent speed. Calculations of WiR were made using the VevoCQ Tool within the Vevolab software (Visualsonics, Toronto, Canada), which is defined as


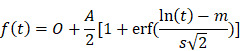


Where 
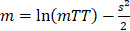
 with the error function *erf* defined as 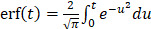. Where *f(t)* represents best-fit function of echo-power; *t* represents time;* A* is amplitude of the plateau and *O* represents offset amplitude. Immediately after bolus perfusion modeling, the destruction-replenishment model was employed. This involves first infusion of microbubbles to achieve a constant flow rate within an ROI, followed by a sudden application of a high powered ultrasound pulse (100% power) to burst the microbubbles, enabling calculation of other perfusion parameters such as mean transit time (mTT) and relative blood volume (rBV). Cine loop recordings in contrast mode were at 11 frame/sec. To determine the perfusion parameters, audio video interleave files for the ROIs were exported then calculated by a nonlinear analysis in GraphPad Prism 7.

**Statistical analysis.** Mixed**-**model regression analysis was performed using the R statistics package to fit the perfusion changes. Statistical significance between two groups was determined using a two-tailed Student *t* test. One-way ANOVA with unequal variances followed by Turkey's multiple comparison test was used to assess significance between multiple groups. All statistical analyses were performed using GraphPad Prism v 7 (GraphPad Software Inc., La Jolla, California). Results were considered statistically significant with a p-value < 0.05.

## Results

**Figure 1:**
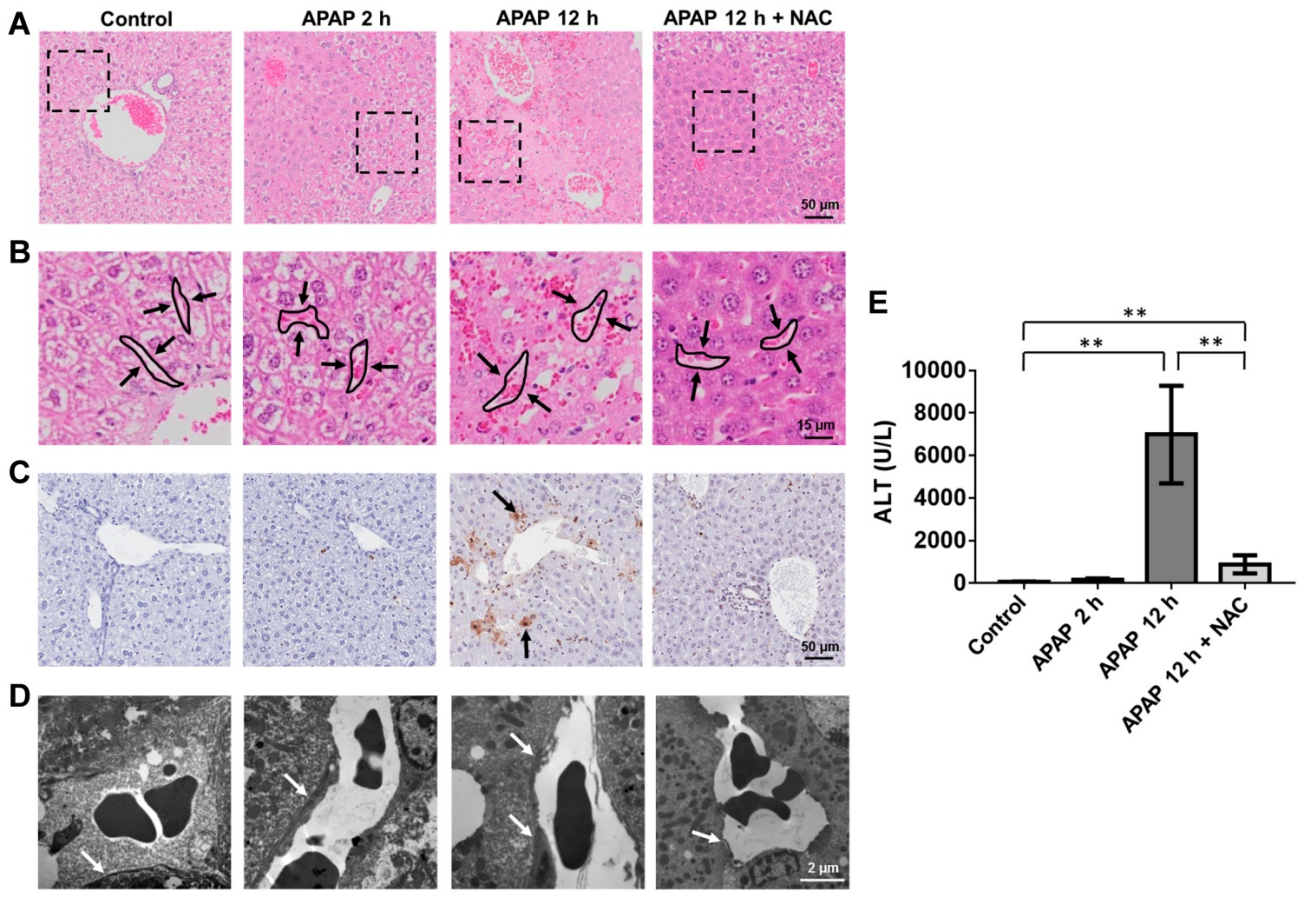
APAP induced damage in the mouse liver. (A). H&E staining of liver sections. Necrosis is observed as pale eosinophilic staining and loss of hepatocyte nuclei. (B). High magnification view of area seen in box of (A) highlighted the sinusoidal area in liver (arrows indicated sinusoidal spaces). Sinusoidal congestion can be seen in livers after 2 h and 12 h APAP overdose as well as the NAC treated group. (C). Representative images of TUNEL stained liver sections in each group. Example of TUNEL positive cells are indicated by black arrows. (D). Transmission electron microscopic images of APAP-related acute sinusoidal endothelial cell injury. Sinusoidal endothelial cells are indicated by white arrows.

**Endothelial cells are an early target of APAP-induced liver injury.** To assess APAP toxicity in liver endothelial cells and hepatocytes, male BALB/c mice were treated with an overdose of APAP at different time points or with NAC treatment after APAP overdose, which is the first choice for treatment of APAP poisoning in the clinic 2, 32. As shown in Figure 1A-B, mild to moderate sinusoidal congestions was observed 2 h after APAP overdose while there was no obvious hepatocyte injury. Similarly, hepatic DNA fragmentation was not detected using TUNEL staining (Figure 1C) at this time point, indicating there was no obvious hepatocyte apoptosis. Furthermore, ALT levels were not significantly increased compared to the control group (Figure 1E). However, sinusoidal endothelial cell swelling was detected by TEM 2 h after APAP administration (Figure 1D), and the endothelial cell injury became more severe at 12 h. There was a significant increase in the number of TUNEL-positive hepatocytes and ALT levels 12 h after APAP overdose. These results suggest that APAP induced sinusoidal endothelial cell injury precedes hepatocyte injury and sinusoidal endothelial cells are an early and direct target for APAP hepatotoxicity.

**Figure 2:**
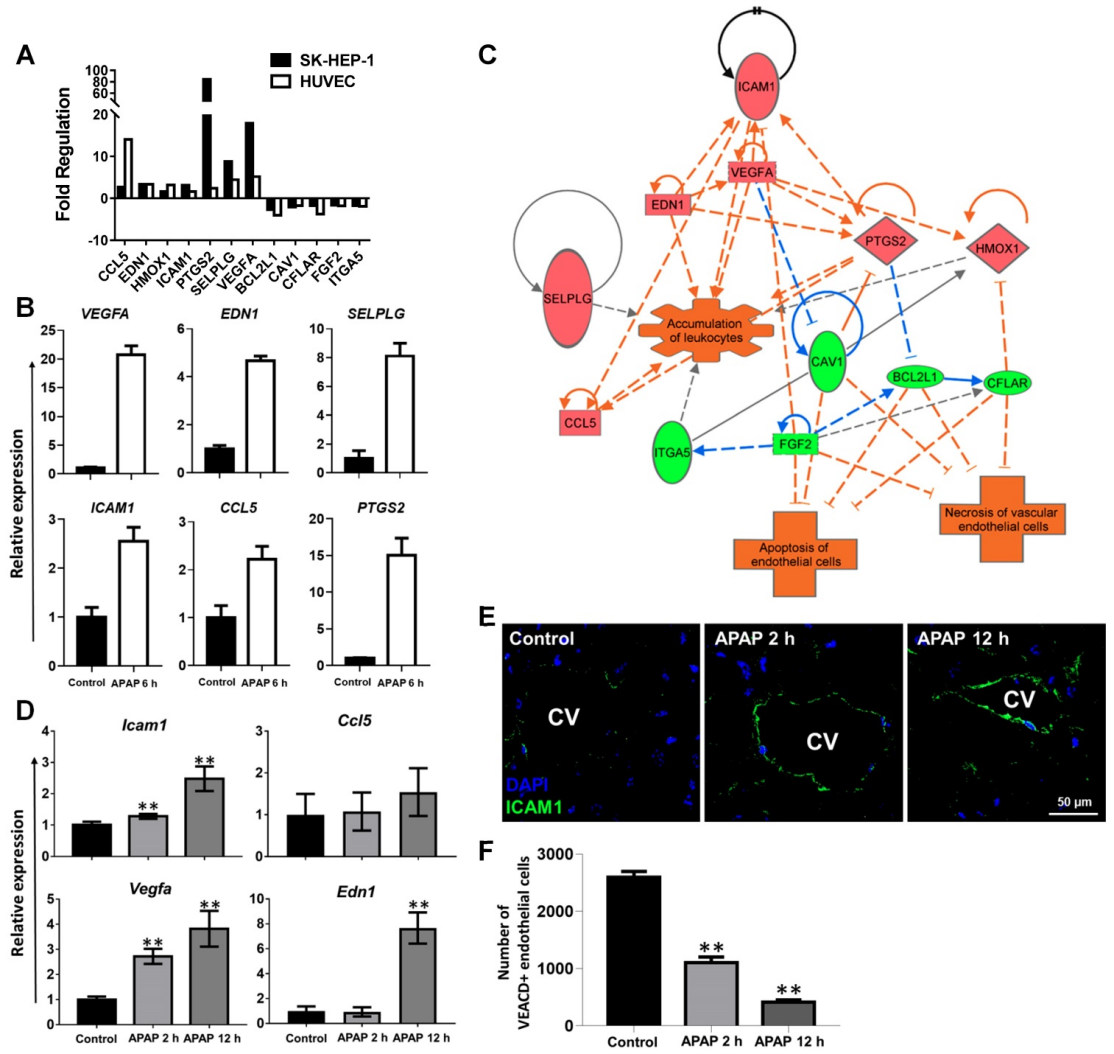
Molecular mechanisms of APAP-induced sinusoidal endothelial cell injury. (A). The commonly up-regulated and down-regulated genes in SK-HEP-1 and HUVEC cells after APAP overdose for 6 h (fold ˃ 1.5, P ˂ 0.05). Data are presented as the mean ± SD (n = 3). (B). qPCR of *VEGFA*, *EDN1*,* SELPLG*,* ICAM1*, *CCL5*, and *PTGS2* mRNAs in SK-HEP-1 cells after APAP overdose for 6 h. (C). IPA of differentially regulated gene networks after APAP overdose. *ICAM1* is involved in the regulation of several genes related to apoptosis, necrosis, and activation of endothelial cells. (D). qRT-PCR of *Icam1*, *Vegfa, Ccl5, and Edn1* mRNA in mouse liver tissues after APAP overdose for 2 and 12 h. (E). The expression of ICAM1 was demonstrated in mouse livers by immunofluorescence staining of ICAM1 (green color) with DAPI (blue color) labelling of the nuclei (CV: central vein; scale bar: 50 μm). (F). Mean number of VECAD^+^CD34^+^CD31^+^Lin^-^ cells isolated from liver tissues were analysed and quantified by flow cytometry. Data are presented as the mean ± SD from 5 independent experiments. **P ˂ 0.01.

**Mechanisms of APAP-induced sinusoidal endothelial cell injury**. A dose-dependent toxicity of APAP was shown in HUVEC and SK-HEP-1 cells (Figure S1) and cell viability decreased as early as 6 h after APAP treatment. Both cell lines treated with 20 mM APAP for 6 and 24 h, reduced the viability of SK-HEP-1 cells to 79% and 35%, and the viability of HUVECs to 57% and 35%, respectively. To define cell signalling pathways involved in the APAP-induced endothelial cell injury, a total of 84 key genes of endothelial cell biology pathways (including vasoconstriction and vasodilation, inflammatory response, cell adhesion, apoptosis and coagulation) were simultaneously assayed using RT^2^ Profiler PCR arrays. SK-HEP-1 and HUVECs were treated with 20 mM APAP for 6 h. Scatter plots of relative mRNA expression for both cell lines before and after APAP treatment are shown in Figure S2. Seven genes were commonly upregulated and 5 genes downregulated in both cells lines after APAP overdose (Figure 2A and Figure S3).

The expression of key genes was validated in both cell lines and primary human hepatic sinusoidal endothelial cells (Figure 2B and Figures S4 and S5A) and mouse liver tissues by qRT-PCR (Figure 2D). ICAM1 and SELPLG within the cellular adhesion molecular pathway, were both significantly up-regulated in two cell lines (Figure 2A-B, p ˂ 0.05). These results indicate that endothelial cells become activated after APAP treatment. APAP overdose also led to increased expressions of CCL5, a chemokine central to leukocyte recruitment, VEGFA, a key regulator of vascular permeability and EDN1 which is involved in regulation of vascular tone 33, 34. Ingenuity Pathway analysis (IPA) was performed to ascertain the cellular context of the differentially expressed genes (Figure 2C) and indicated APAP induced leukocyte accumulation and endothelial cell damage. These results suggest ICAM plays a prominent role in the endothelial cells response to APAP. Importantly, analysis of gene expression of human livers using a public microarray database showed that there was a 7 fold increase in EDN1 expression and an overall 1.6 fold increase in ICAM expression after APAP overdose (Figure S5B), which were consistent with our *in vitro* and *in vivo* results.

To visualise overall ICAM1 expression in APAP overdose animals, liver sections were stained with ICAM1 antibody (Figure 2E). Enhanced ICAM1 expression was found in the centrilobular regions of mouse livers after APAP overdose, further demonstrating the induction of endothelial adhesion molecules. We next examined the impact of APAP overdose on the endothelial cell-specific adhesion protein VE-cadherin (VECAD), which plays an important role in endothelial cell biology through control of the cohesion and organisation of the intercellular junctions 35. The number of VECAD positive endothelial cells decreased significantly 2 h after APAP overdose, and was further depleted at 12 h (Figure 2F, **P ˂ 0.01). Taken together, the results indicate that APAP overdose resulted in the activation of endothelial adhesion pathways and vascular damage evidenced by opening of the endothelial junctions, inflammatory cell homing, increased microvasculature permeability and vascular leakage.

**Figure 3:**
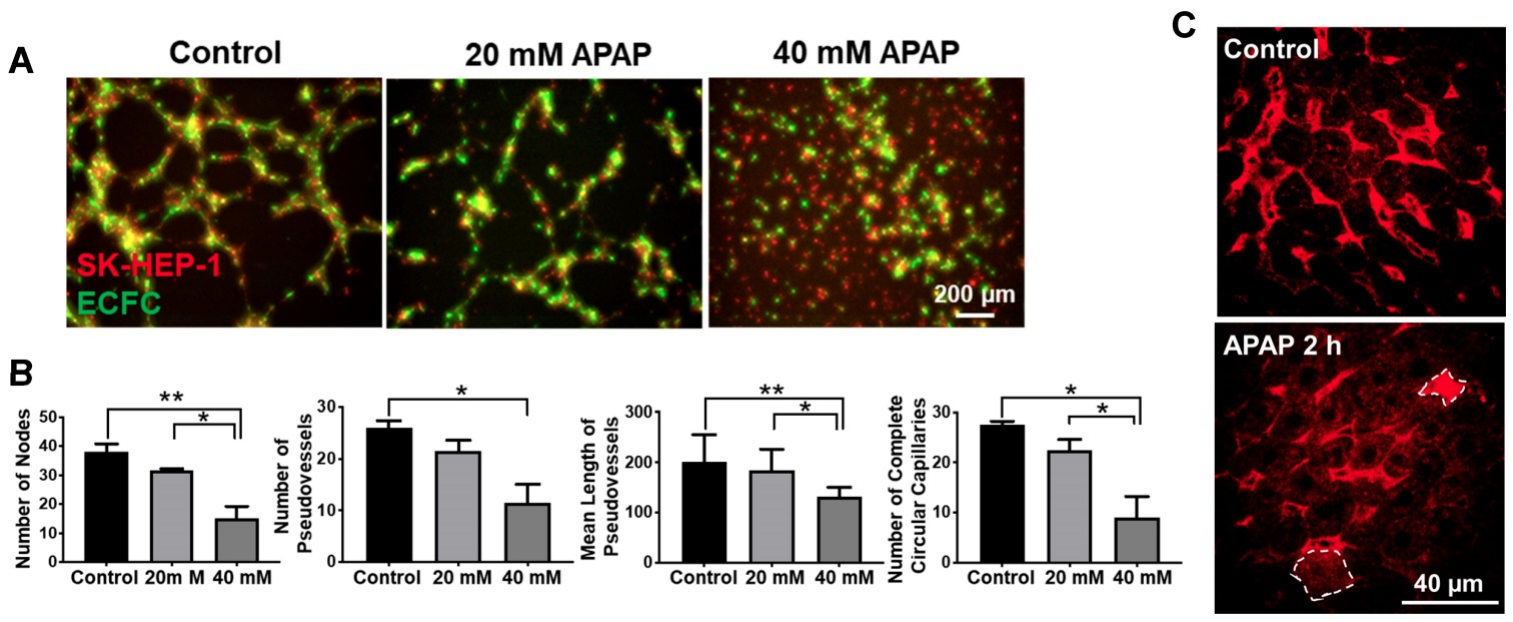
APAP overdose affected vascular tube formation and vascular integrity in the liver. (A). 2D vascular tube formation assay using ECFC cells (green) and SK-HEP-1 cells (Red) either untreated and treated with various concentrations of APAP. Images represent t = 12 h, scale bar: 200 μm. (B). Quantitative analysis of vessel morphometry, including number of nodes, number of vessels, mean length of vessels, and number of complete circular capillaries. *P ˂ 0.05, **P ˂ 0.01; one-way ANOVA. (C) Multiphoton microscopy imaging of mouse liver after intravenous injection of DXR70. Red color represents DXR fluorescence in the liver and circled cells (dashed lines) indicate DXR uptake by hepatocytes. Scale: 40 μm.

**APAP overdose effects on vascular tube formation and vascular integrity.** As the level of ICAM1 is related to vascular tube formation 36, we further evaluated the effects of APAP on the formation of pseudo-vessels by SK-HEP-1 and HUVECs on 2D Matrigel. Pseudo-vessel networks were sufficiently formed by mixed SK-HEP-1 cells and human endothelial colony forming cells (ECFC) as well as HUVEC and ECFC (Figure 3A and Figure S6A, Video S1&2). Cells treated with 40 mM APAP did not form pseudo vessels but cells treated with 20mM APAP commenced vessel formation after 12 h (Figure 3A and Figure S6A, Video S3-6). To gain further insight into the vessel morphometry after APAP treatment, we quantified the number of pseudovessels, nodes and complete circular capillaries as well as the mean length of pseudovessels. Large numbers of vessels were lost after 20mM APAP treatment and the node number was significantly less than control groups (Figure 3B and Figure S6B). All these findings indicated APAP overdose impaired vascular tube formation of endothelial cells.

VECAD positive endothelial cells decreased in mouse livers after APAP treatment for 2h (Figure 2F), indicating opening of the endothelial junctions and vascular leakage. Then we examined the sinusoidal integrity by intravenous injection of DXR70 and imaged the liver by multiphoton microscopy according to the reported method 37. DXR70 was evenly distributed in sinusoids but was not taken up by hepatocytes in the control liver, while tapered sinusoids were shown in the APAP treated liver and DXR70 fluorescence was found in a few hepatocytes (Figure 3C). These results not only reflected compromised plasma membranes and sinusoidal vascular leak at the early phase of APAP overdose, but also indicated the injury of hepatocytes which do not show pathological changes.

**Figure 4:**
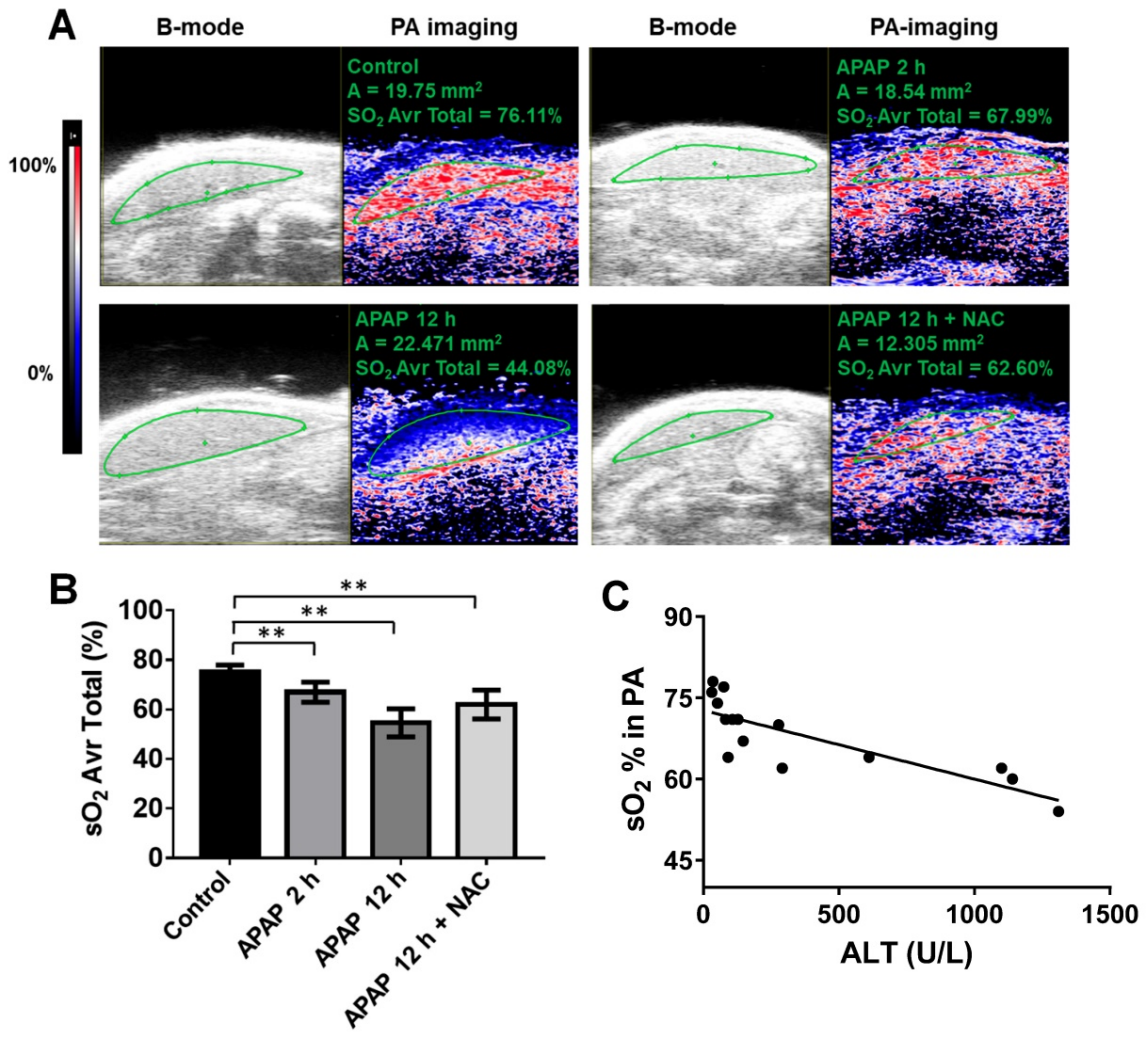
APAP overdose and NAC treatment effect on oxygen saturation of the liver. (A). Representative PA images (right panel) co-registered with greyscale B mode imaging (left panel) of livers in each group. The heat map represents oxygen saturation levels ranging from 100 % (red) to 0% (dark blue). sO_2_ Avr total represents the oxygen saturation calculated by percentage of oxygenated haemoglobin in relation to total haemoglobin. (B). The average oxygen saturation of each group detected by PA imaging (n = 5). (C). Relationship between the levels of oxygen saturation of livers and ALT levels in control, APAP 2 h, and APAP+NAC groups (R^2^ = 0.67, P = 0.0002, n = 15).

**APAP overdose effects on oxygen levels in the liver.** Based on our findings above, we further explored potential imaging-based vascular-related biomarkers for early detection of APAP-induced liver injury using ultrasound. First, PA imaging was performed on the left lobe of the mouse liver, and relative oxygen saturation levels were plotted over time. Heat-map images of oxygen saturation of the liver, co-registered with greyscale B mode ultrasound, of representative mice from each group are shown in Figure 4A. Levels of hepatic oxygen saturation were associated with APAP-induced liver injury (Figure 4A). Oxygen saturation of the liver parenchyma was significantly decreased to a mean of 64 % after APAP overdose for 2 h and 56 % after APAP overdose for 12 h compared to control liver of over 75 % (Figure 4B). NAC treatment improved oxygen saturation to approximately 63 %.

We then assessed how oxygen saturation may reflect degrees of liver injury and found that oxygen saturation levels of livers correlated well with the ALT level from early stage of APAP overdose in the blood (R = 0.67, p = 0.0002) (Figure 4C) but not the late stage (this correlation only include control, 2 h APAP overdose, and NAC treated groups). Significant changes in hepatic oxygen saturation were detected as early as 2 h after APAP overdose, which preceded ALT changes. Thus, we suggest that oxygen saturation can be used as a biomarker for early or mild APAP-induced liver injury especially when ALT levels are normal.

**Figure 5:**
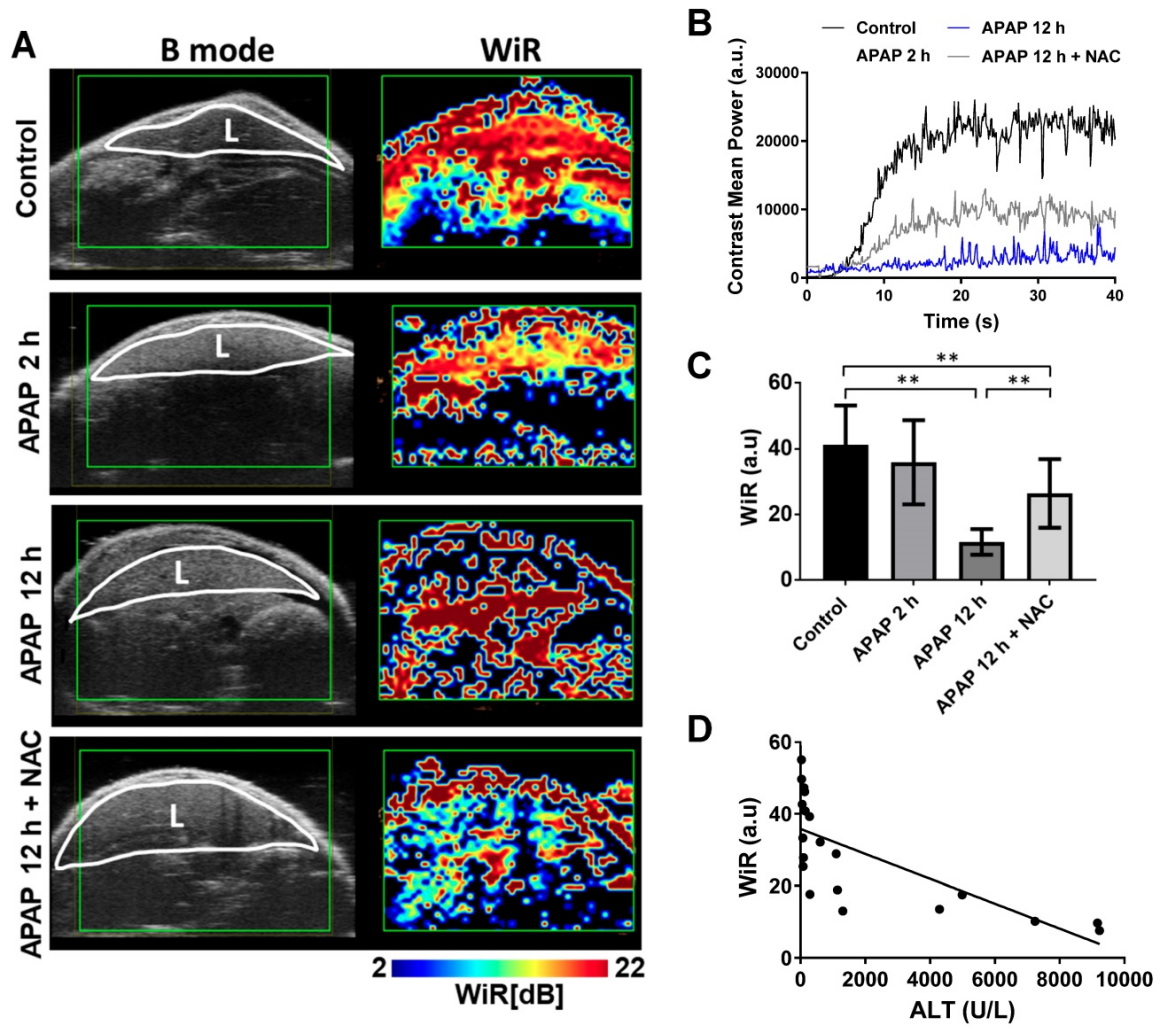
APAP overdose and NAC treatment effect on wash-in rate (WiR) of the liver.** (**A). Left panel of images (B mode) represent mouse liver obtained using normal ultrasound to indicate liver anatomy (L: liver). High resolution parametric perfusion maps of the regional liver (Right panel). Livers of control, APAP overdose for 2 h and 12 h, and NAC treatment are displayed in pseudocolor. Red and orange colors represent the higher WiR, while blue color represents the lower WiR. After APAP overdose for 2 h, the red color changed to yellow and green in the liver. A noticeable blue band is present after APAP overdose for 12 h while NAC treated liver was less blue and more green and yellow. (B). Representative single liver perfusion tracings were obtained for the control, APAP overdose for 2 h and 12 h, and NAC treated animals, by monitoring initial filled nonlinear signals of intact microbubbles. (C). The average WiR of each group based on nonlinear contrast imaging (n = 5). (D). Relationship between the WiR and ALT levels in each group (R^2^ = 0.53, P = 0.0003, n = 20).

**Figure 6:**
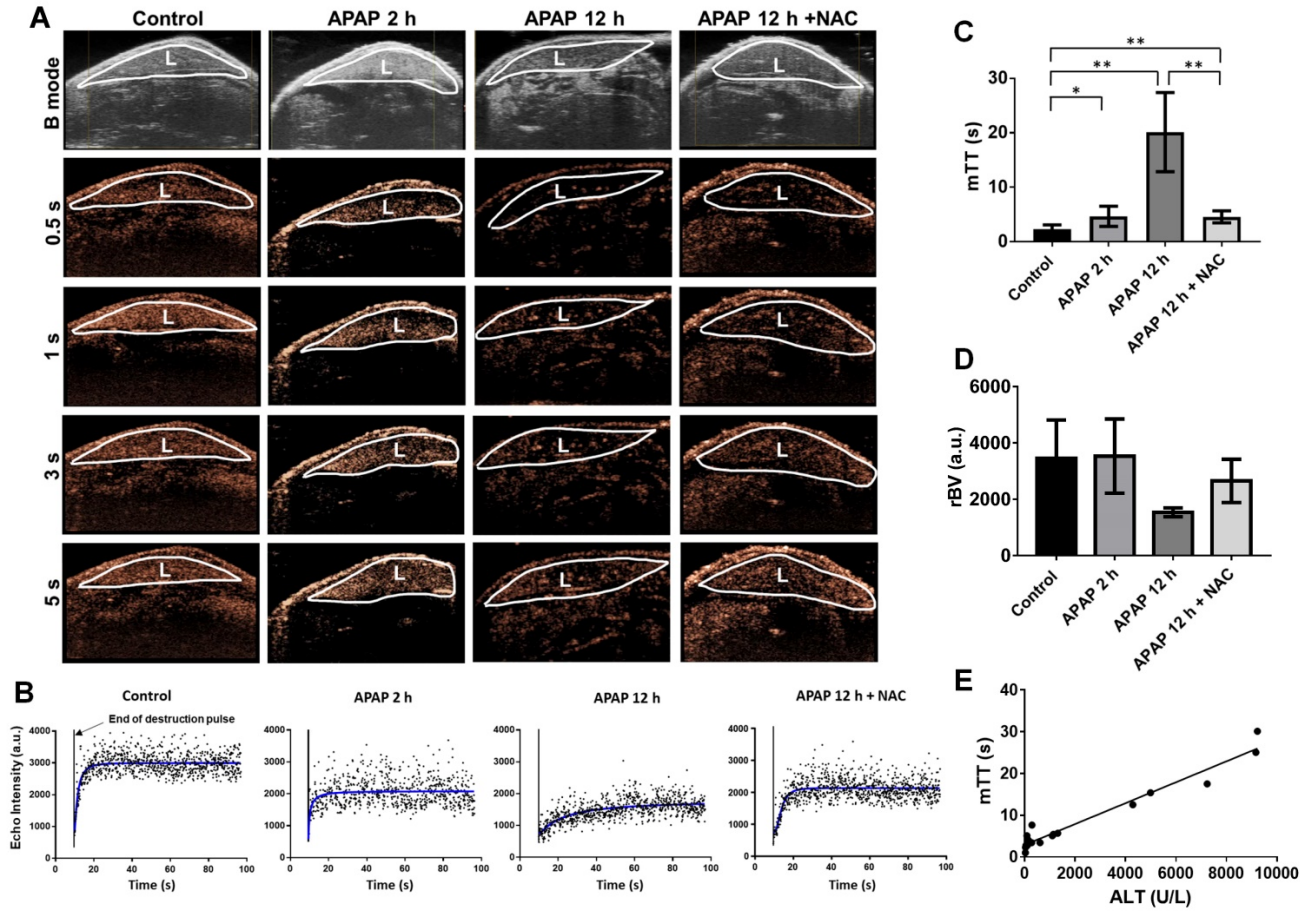
APAP overdose and NAC treatment effect on mean transit time (mTT) of the liver perfusion. (A). Nonlinear contrast imaging sequence at various time points of refilled microbubbles after “burst” at the regional area of the liver in each group. Top panel of images (B mode) represent the mouse liver obtained using normal ultrasound to indicate liver anatomy (L: liver). By 0.5 s, livers of control and APAP overdose for 2 h started to refill with microbubbles. After 1 s, control liver was almost fully refilled by microbubbles. By 5 s, livers of APAP overdose for 2 h and NAC treatment were fully refilled by microbubbles, while the plateau of liver perfusion was not reached in the liver of APAP overdose for 12 h. (B). Representative liver perfusion curves based on the sequence of refilled microbubbles after a high mechanical index “burst”. Bursts are indicated by arrows. The dots are linearized signals recorded by contrast imaging and the curve was fitted by the Destruction-replenishment perfusion model. (C). The average mTT of each group based on nonlinear contrast imaging and calculated by the destruction-replenishment model. Data are presented as the mean ± SD (n = 5, **P ˂ 0.01). (D). The average rBV of each group. Data are presented as the mean ± SD (n = 5).** (**E). Relationship between the mTT and ALT levels in each group (R^2^ = 0.9527, P ˂ 0.0001, n = 20).

**APAP overdose effects on hepatic microcirculation.** Immediately after photoacoustic imaging, contrast-enhanced ultrasound was performed to assess hepatic perfusion. Obvious effects of APAP overdose on perfusion parameters were observed using both bolus and destruction-replenishment perfusion models (Figure 5 and 6). High resolution parametric perfusion maps were developed to visualise and monitor microvascular perfusion changes in space and time, providing insights into the distributional changes in the microcirculation of the injured liver induced by APAP overdose. Real-time perfusion imaging clearly showed the decreased wash-in rate (WiR) of the initial contrast microbubbles in the APAP overdose treated liver (Figure 5A and Video S7-10), as presented in pseudo colour (the colour changed to blue and dark blue). The parametric maps revealed the WiR in the liver decreased as early as 2 h post APAP overdose and was more obviously reduced after 12 h. In NAC treated mice, delayed perfusion recovery persisted in the liver. These parametric perfusion images reflected rapid and marked reduction in the microvascular perfusion of the liver after APAP overdose. Representative single liver perfusion tracings were obtained for the control, 2 h and 12 h APAP overdose, and NAC treated animals, by monitoring initial filled-induced nonlinear signals of intact microbubbles (Figure 5B). WiR was significantly longer in the liver 12 h after APAP overdose compared to the control group (Figure 5C), indicating that APAP overdose decreased the blood flow velocity in the liver. In contrast, NAC treated livers showed obvious improvement in WiR compared to 12 h APAP overdose.

To further evaluate the hepatic microcirculation, the destruction-replenishment perfusion model was performed (Figure 6 and Video S11-14). Representative nonlinear contrast images of the liver after acutely collapsing microbubbles with an ultrasound burst and the refilled microbubbles were monitored (Figure 6A). By 0.5 s, the liver started to refill with microbubbles. After 1-2 s, the plateau phase of perfusion was reached in the control liver, whereas the hepatic perfusion 2 h after APAP overdose and in NAC treatment groups returned to levels close to baseline by about 5 s. The refilled microbubbles of 12 h after APAP overdose reached the flat phase over 10 s. The representative single hepatic perfusion tracings were obtained by monitoring refill induced nonlinear signals of intact microbubbles after immediately collapsing by ultrasound burst (Figure 6B). The imaging signals from slight breathing motion artefacts were digitally subtracted from the initial frames. The perfusion parameter, mTT, represents the microbubbles transit time in the regional liver and was determined by the destruction-replenishment perfusion model based on a curve fitting of data in Figure 6B. Compared to control liver, APAP overdose significantly decreased hepatic perfusion at 2 h, where mTT was significantly extended at 2 h and further increased at 12 h after APAP overdose (Figure 6C). After NAC treatment, hepatic perfusion was improved and mTT was significantly shorter compared to untreated 12 h APAP group. On the other hand, relative blood volume (rBV) quantified using this model (Figure 6D) showed that although rBV of the liver after APAP overdose for 12 h is less than normal liver, it is not statistically significant. This parameter is therefore not as sensitive as mTT and WiR. Furthermore, WiR correlated slightly with ALT levels (Figure 5D, R = 0.53, P = 0.0003), while mTT showed a significant correlation with ALT levels (Figure 6E, R = 0.9527, P ˂ 0.0001), suggesting these imaging biomarkers accurately detect the liver injury induced by APAP overdose at the early phase and reflect the treatment response of injured livers. Importantly, mTT is the optimal imaging marker for early detection of APAP induced liver injury.

## Discussion

It is imperative to discover and validate more sensitive and specific translational biomarkers of APAP-induced liver injury. Most studies of APAP hepatotoxicity have focused on the hepatocellular injury 38, and current hepatocyte-related biomarkers have delayed presentation time and a lack of sensitivity, remaining a critical impediment to the treatment of APAP overdose 9. Although studies have shown APAP is also toxic to endothelial cells and precedes the injury of hepatocytes 9-11, 39, the mechanisms involved are still unclear. This study revealed the detailed mechanisms of APAP-induced endothelial cell injury. Our data also provide evidence that imaging-based vascular-related markers are able to detect early and mild APAP-induced liver injury.

By profiling gene expression in endothelial cells after APAP overdose, we have shown that ICAM1 is at the apex of signalling pathways activated in endothelial cells during APAP hepatotoxicity. ICAM1 is an intercellular adhesion molecule continuously present in membranes of endothelial cells 40. ICAM1 and SELPLG, which were shown to be upregulated in both cells response to APAP, are involved in the activation of endothelial cells and binding of leukocytes to endothelial cells during leukocyte docking and transmigration through the endothelial cell layer 33. A few studies have shown that ICAM-1 expression was increased in mouse livers after APAP administration for 10 h and the marked protection against APAP hepatotoxicity was found in ICAM-1-deficient mice. But these studies demonstrated APAP overdose could induce the increase of ICAM-1 in tissues at the late or severe phase of liver injury, which was associated with the accumulation of neutrophils 41, 42. This is the first study to show the upregulated ICAM-1 expression of human endothelial cells in direct response to APAP overdose at the early phase. Subsequent experiments showed that higher expression of ICAM1 was found in endothelial cells around the hepatic central vein as early as 2 h after APAP overdose, which is consistent with APAP induced hepatotoxicity initially occurring in the areas adjacent to the central vein 43. Along with these changes there was up-regulation of chemokines involved in leukocyte recruitment (CCL5) and growth factors involved with vascular tone (EDN1) 34. Analysis of human genes of primary human hepatic sinusoidal endothelial cells and patient tissues after APAP overdose induced acute liver failure showed that ICAM1 and EDN1 obviously increased, further supporting our findings. This is the only available public dataset of liver gene expression after APAP overdose in comparison to healthy liver. All these results suggest induction of vascular damage and an inflammatory response, leading to opening of the endothelial junctions and inflammatory cell recruitment.

VECAD is an endothelial-specific cadherin that plays an important role in cohesion and organisation of intercellular junctions 35, 44. The depletion of VECAD positive endothelial cells further suggests APAP overdose could result in the opening of the endothelial junctions, increased vascular permeability and vascular leakage. We also confirmed that APAP overdose impaired the functions of endothelial cells, such as vascular tube formation and vascular integrity, at the early stage of liver injury. Therefore, endothelial cells are an early target of APAP hepatotoxicity and vascular related biomarkers are potentially useful for early diagnosis of APAP induced liver injury. Hepatic perfusion may mirror the main consequences of APAP overdose induced liver injury at the early stage, which can be characterised by impairment of hepatic microcirculation and liver tissue oxygenation. Although standard imaging techniques allow the structural investigation of the vascular consequences of liver injury, the hepatic microcirculation is not easily assessed and monitored. Noninvasive measurement of hepatic blood flow by Doppler is not sensitive or accurate 45 and evaluation relies on indirect methods such as indocyanine green 46 and galactose clearance test. Taken together, new techniques capable of giving reliable information on liver microcirculation are still needed.

Therefore, using advanced ultrasound technologies including PA and CEU imaging, we have demonstrated that APAP overdose resulted in a reproducible pattern of injury to the hepatic microcirculation. By PA imaging, oxygenation saturation of the liver could be evaluated based on measurements of oxygenated and deoxygenated haemoglobin of the tissue *in vivo* 23. Oxygenation saturation of the liver was found to be significantly reduced from 2 h after APAP overdose. The changes in oxygen content of blood vessels indicate the changes in blood flow and circulation 47. In recent years, CEU has been proposed as a tool for measuring real-time functional aspects of microvasculature in biological tissues 46-49. Microbubbles can be used as contrast agents to generate nonlinear signals during CEU imaging, allowing real-time recording and analysis in all cases. We applied microbubbles as contrast agents to systematically investigate the effect of APAP overdose on hepatic microcirculation. Our results show that impaired hepatic microcirculation can be detected as early as 2 h after APAP overdose using CEU. We showed that not only was there a delay in reaching peak blood flow in the liver after APAP overdose, but overall reduced blood volume and blood flow (reflected by WiR) were also found in the liver. NAC treatment, which is shown to reduce the hepatotoxicity of APAP, significantly improves the conditions of hepatic microcirculation. These results suggest alterations in liver microperfusion occur in APAP-induced liver injury and potentially that these microcirculatory changes are important in injury progression and recovery. Importantly, the damage to hepatic microcirculation has been shown to precede evidence of hepatocyte necrosis and apoptosis. A further benefit of CEU is that there are no adverse effects related to the procedure 9. Thus, we believe that CEU is a feasible novel, quantitative, and noninvasive technique for evaluating hepatic microcirculation for assessing APAP toxicity in clinic.

The vascular related imaging biomarkers correlated well with a standard plasma biomarker, ALT 50. A parameter of oxygenation saturation correlated well with early and mild liver injury but may not reflect late or severe liver injury. This may be due to the oxygenation measurements, where haemoglobin is not present at a significant level once the tissue is necrotic or poorly vascularised 23. Hence, this parameter is more suitable for detecting early or mild liver injuries, especially those with normal ALT levels. WiR, which is determined by a bolus infusion model, showed a moderate correlation with ALT levels. WiR was affected by the injection rate, which may also limit the use of this parameter as a precision imaging biomarker. Our study showed that mTT had the best correlation with ALT levels compared to other parameters. It is noted that mTT was significantly prolonged 2 h after APAP overdose, while ALT levels did not significantly increase, indicating mTT can accurately evaluate the liver injury both at early phase and late phase. Overall, mTT was more reliable and gave a more comprehensive depiction of APAP induced liver injury at both early and mild as well as late phases of liver injury. We suggest this vascular related imaging biomarker also can be applied to monitor and evaluate microvascular dysfunction such as ischemia reperfusion 51, 52.

In conclusion, this study investigated the mechanisms of APAP overdose induced microcirculatory injury and endothelial cell damage for the first time. Systematic evaluation of the hepatic microcirculation after APAP overdose by ultrasonography showed that hepatic microvascular disturbance correlates well with the severity of APAP hepatotoxicity and precedes the necrosis of hepatocytes. Of all evaluated parameters, mTT by ultrasonography has better reproducibility and reliability, and could serve as a noninvasive biomarker for the diagnosis and treatment evaluation of APAP overdose. With further development, this novel technique can be translated to clinical use to improve the assessment of liver injury and the speed of clinical decision-making.

## Supplementary Material

Supplementary figures and tables.

Video S1.

Video S2.

Video S3.

Video S4.

Video S5.

Video S6.

Video S7.

Video S8.

Video S9.

Video S10.

Video S11.

Video S12.

Video S13.

Video S14.
