# Supplementary material for: Imaging-based vascular-related biomarkers for early detection of acetaminophen-induced liver injury
Source: Theranostics. 2020 May 18;10(15):6715–27. doi: 10.7150/thno.44900 (PMC7295051; doi:10.7150/thno.44900)
Supplement: Supplementary file 1 — Supplementary figures and tables. [file thnov10p6715s1.pdf]

Supplementary tables

Table S1. Sequences of primers used for validation of gene expression in human cells

Gene	Primer	Nucleotide sequence (5' to 3')
VEGFA	Forward	GGAGGAGGGCAGAATCATCAC
	Reverse	CTTGGTGAGGTTTGATCCGC
ICAM1	Forward	GCAACCTCAGCCTCGCTAT
	Reverse	TGACTTTTGAGGGGGACACAG
EDN1	Forward	CCAGAAACAGCAGTCTTAGGC
	Reverse	TCACGGTCTGTTGCCTTTGT
SELPLG	Forward	TTGTCACTAAAGCAGAGAAGCC
	Reverse	GAGTGGTGTCAGTGCTGTTC
CCL5	Forward	CTCGCTGTCATCCTCATTGCT
	Reverse	AGCACTTGCCACTGGTGTAG
HPRT	Forward	TCAGGCAGTATAATCCAAAGATGGT
	Reverse	AGTCTGGCTTATATCCAACACTTCG

Table S2. Sequences of primers used for validation of gene expression in mouse tissues

Gene	Primer	Nucleotide sequence (5' to 3')
Vegfa	Forward	AGAAGGAGAGCAGAAGTCCCA
	Reverse	CTTTGGTGAGGTTTGATCCGC
Icam1	Forward	CAGTCCGCTGTGCTTTGAGAA
	Reverse	GAAATTGGCTCCGTGGTCCC
Edn1	Forward	GGACATCATCTGGGTCAACA
	Reverse	GCTTGGACCTGGAAGAACCT
Ccl5	Forward	CCTGCTGCTTTGCCTACCTCTC
	Reverse	ACACACTTGGCGGTTCCTTCGA
Hprt	Forward	CCCCAAAATGGTTAAGGTTGC
	Reverse	AACAAAGTCTGGCCTGTATCC

Supplementary Figures

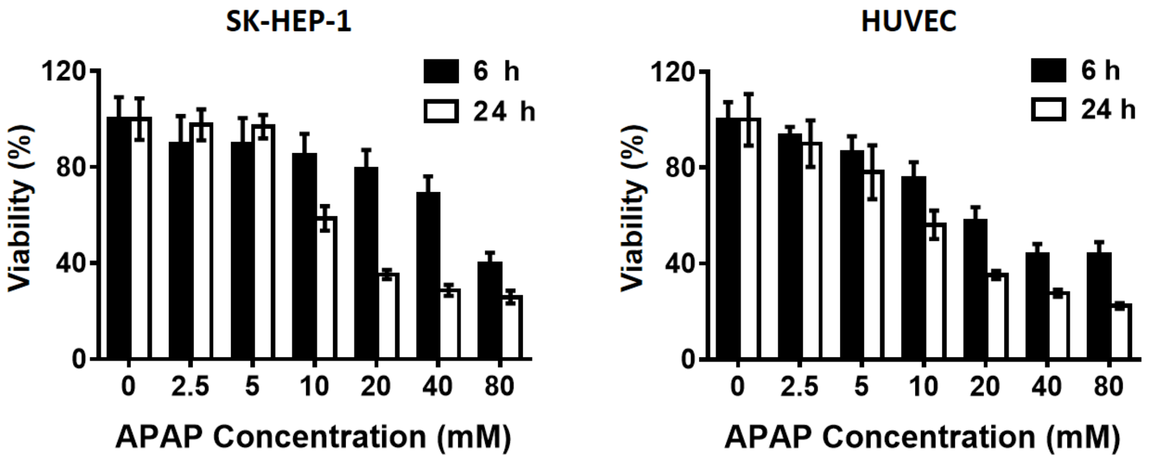

Figure S1. MTT assay of SK-HEP-1 and HUVEC cells after treatment with various concentrations of APAP for 6 h or 24 h. Data are presented as the mean ± SD (n = 3).

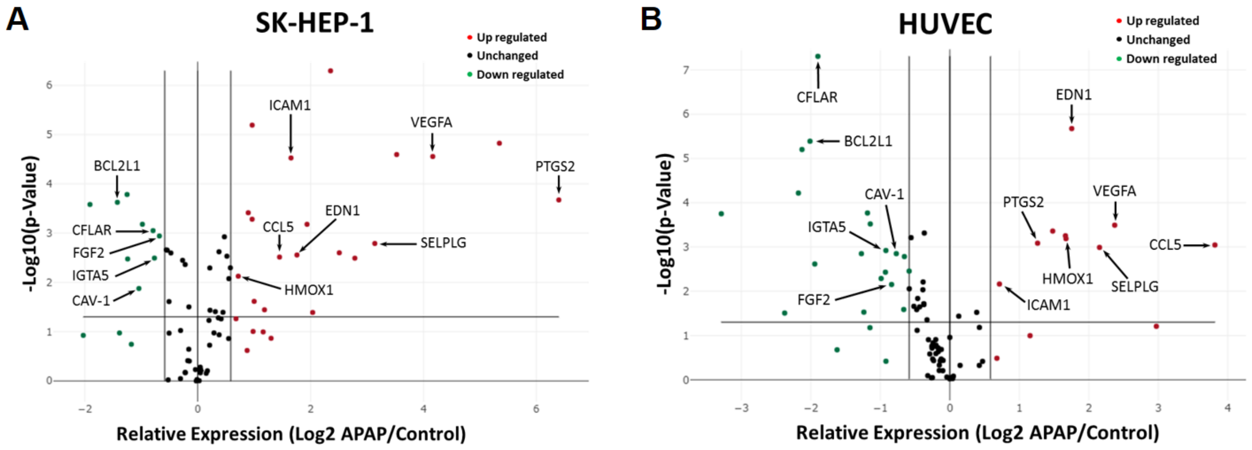

Figure S2. Relative expression of endothelial markers after APAP overdose. Scatter plot displays fold change and significance between control and APAP treated SK-HEP-1 cells (A) or HUVEC cells (B), p values are calculated based on a Student's t-test of the replicate 2^ (-Delta CT) values. The y-axis shows the negative log10 of p-values and the x-axis is the difference in mRNA expression between the two groups as log2 fold changes. Vertical sliders demarcate either up- or down- regulation above a 1.5 fold change. Horizontal sliders demarcate a p-value of < 0.05. Genes with altered expression levels more than 1.5 fold with a p-value of < 0.05 in both cell lines are indicated by arrows.

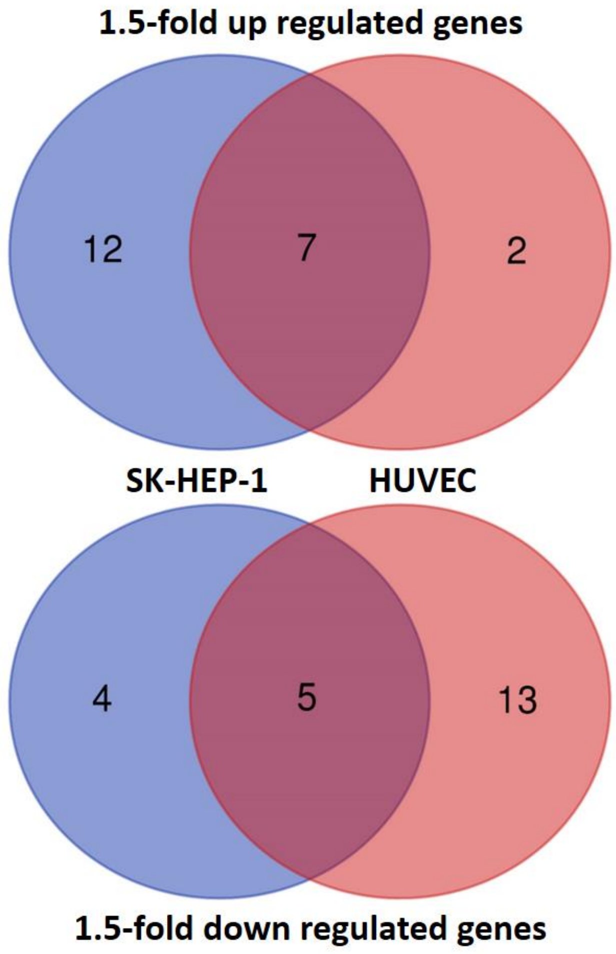

Figure S3. Venn diagrams show number of 1.5 fold up-regulated and down-regulated genes after APAP overdose for 6 h in SK-HEP-1 and HUVEC cells.

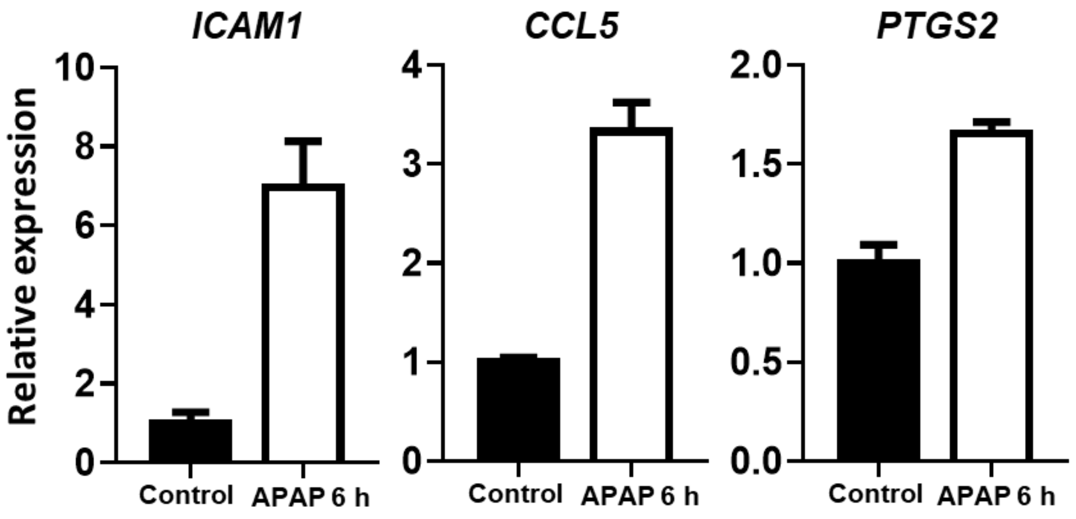

Figure S4. qRT-PCR analysis of *ICAM1*, *CCL5*, and *PTGS2* mRNA expression in HUVEC cells after APAP overdose for 6 h.

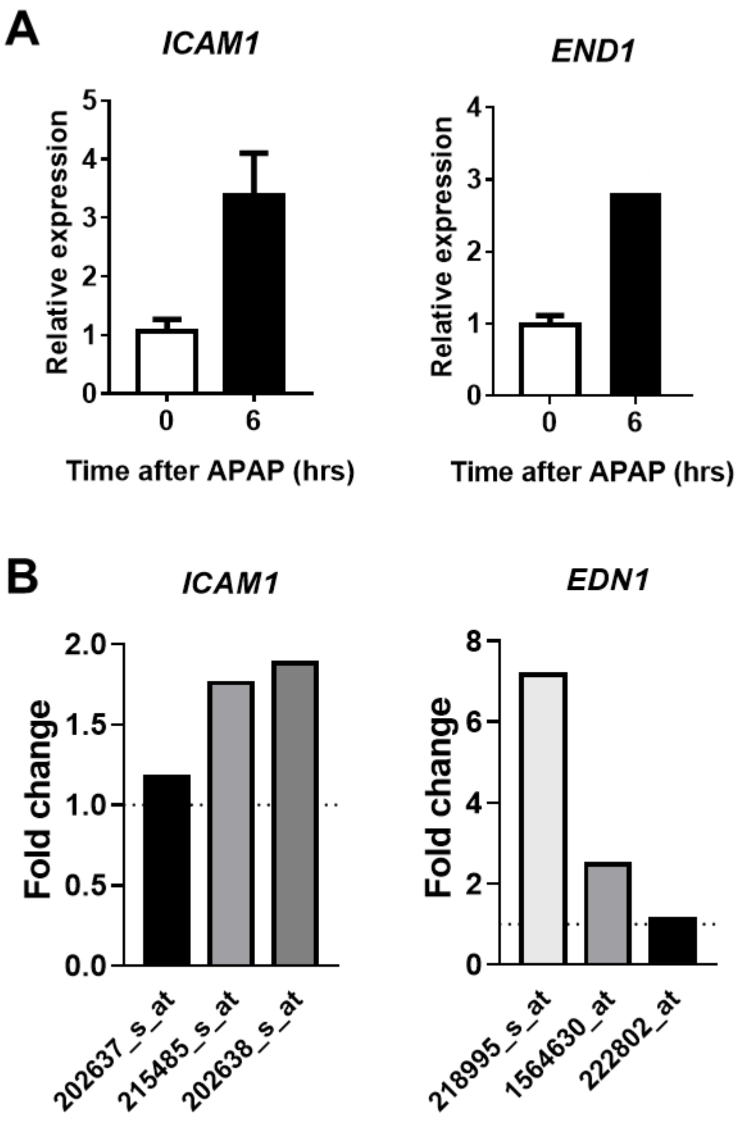

Figure S5. Relative expression of gene expression after APAP overdose in human primary hepatic sinusoidal endothelial cells and human tissue samples using a public dataset. (A). qRT-PCR analysis of *ICAM1* and *EDN1* mRNA expression in primary human hepatic sinusoidal endothelial cells after APAP overdose for 6 h. (B). Fold change in mRNA expression after APAP overdose relative to healthy livers was calculated using the GSE74000 dataset for three different probe sets for ICAM and EDN1. X-axis represents probe set ID in dataset.

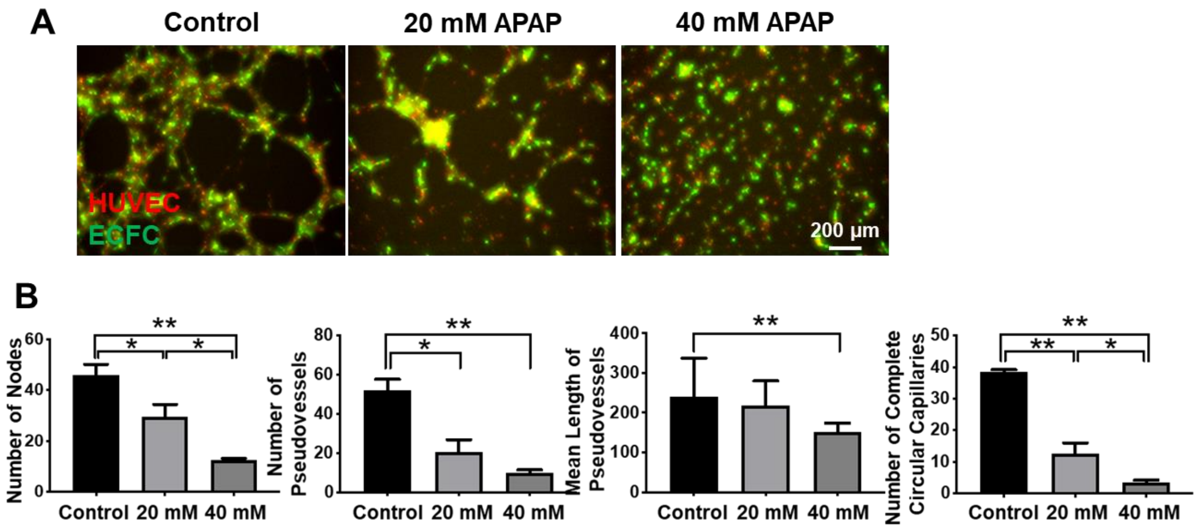

Figure S6. Vascular tube formation and vascular integrity. (A). 2D vascular tube formation assay for mixed ECFC cells (green) and HUVEC cells (red) either untreated and treated with various concentrations of APAP. Images represent t = 12 h, scale bar: 200 μm. (B). Quantitative analysis of vessel morphometry, including number of nodes, number of vessels, mean length of vessels, and number of complete circular capillaries. Data are presented as the mean ± SD (n = 5), **P* < 0.05, ***P* < 0.01; one-way ANOVA.

Supplementary Video

Video S1. Cooperation of mixed ECFC cells (green) and control SK-HEP-1 cells (red) to form pseudovessels. Tube formation was monitored by Olympus IX81 Live Imaging Microscope. Time-lapse acquisitions were performed every 30 min up to 12h.

Video S2. Cooperation of mixed ECFC cells (green) and APAP 20 mM treated SK-HEP-1 cells (red) to form pseudovessels. Tube formation was monitored by Olympus IX81 Live Imaging Microscope. Time-lapse acquisitions were performed every 30 min up to 12h.

Video S3. Cooperation of mixed ECFC cells (green) and APAP 40 mM treated SK-HEP-1 cells (red) to form pseudovessels. Tube formation was monitored by Olympus IX81 Live Imaging Microscope. Time-lapse acquisitions were performed every 30 min up to 12h.

Video S4. Cooperation of mixed ECFC cells (green) and control HUVEC cells (red) to form pseudovessels. Tube formation was monitored by Olympus IX81 Live Imaging Microscope. Time-lapse acquisitions were performed every 30 min up to 12h.

Video S5. Cooperation of mixed ECFC cells (green) and APAP 20 mM treated HUVEC cells (red) to form pseudovessels. Tube formation was monitored by Olympus IX81 Live Imaging Microscope. Time-lapse acquisitions were performed every 30 min up to 12h.

Video S6. Cooperation of mixed ECFC cells (green) and APAP 40 mM treated HUVEC cells (red) to form pseudovessels. Tube formation was monitored by Olympus IX81 Live Imaging Microscope. Time-lapse acquisitions were performed every 30 min up to 12h.

Video S7. Real-time imaging of WiR in control liver.

Video S8. Real-time imaging of WiR in the liver of APAP overdose for 2 h.

Video S9. Real-time imaging of WiR in the liver of APAP overdose for 12 h.

Video S10. Real-time imaging of WiR in the liver of APAP overdose and NAC treatment.

Video S11. Real-time imaging of refilled microbubbles after “burst” at the regional area of the control liver.

Video S12. Real-time imaging of refilled microbubbles after “burst” at the regional area of the liver after APAP overdose for 2 h.

Video S13. Real-time imaging of refilled microbubbles after “burst” at the regional area of the liver after APAP overdose for 12 h.

Video S14. Real-time imaging of refilled microbubbles after “burst” at the regional area of the liver after APAP overdose and NAC treatment.
